# Flow cytometric features of lymphoid subsets in healthy and diseased cats

**DOI:** 10.3389/fvets.2025.1640229

**Published:** 2025-08-01

**Authors:** Cesar Guillermo Meneses-Nava, Alessandra Ubiali, Greta Dossi, Sabine E. Hammer, Barbara C. Rütgen, Chiara Locatelli, Angelica Stranieri, Valeria Martini

**Affiliations:** ^1^Department of Veterinary Medicine and Animal Sciences, University of Milan, Lodi, Italy; ^2^Immunology, Department of Biological Sciences and Pathobiology, University of Veterinary Medicine, Wien, Austria; ^3^Clinical Pathology, Department of Biological Sciences and Pathobiology, University of Veterinary Medicine, Wien, Austria

**Keywords:** flow cytometry, peripheral blood, lymphocytes, FSC, MFI

## Abstract

**Introduction:**

Flow cytometry (FC) is widely used in humans and dogs to diagnose and characterize hematopoietic neoplasms. Conversely, its use in feline patients is still limited, leading to a lack of standardized protocols and subjective data interpretation.

**Methods:**

Herein, we describe FC features of circulating lymphoid subsets in a total of 20 cats: 9 healthy cats, 6 diseased cats without hematopoietic neoplasm, and 5 cats with probable chronic lymphocytic leukemia (CLL), using a panel of 10 antibodies and a multicolor approach, in terms of both cell size (nFSC) and degree of antigen expression (MFI).

**Results:**

Three main subsets were identified in healthy cats and diseased cats without hematopoietic neoplasm (namely, CD5 + CD45R-, CD21 + CD45R + and CD5 + CD45R+). CD4 + CD8- cells outnumbered CD4- CD8 + cells. Low percentages of CD4 + CD8 + and CD134 + cells were also present. MHCII had higher fluorescence intensity in B- than in T-cells. CD9 was not expressed on leukocytes surface, but on small events possibly referable to platelet clumps. In diseased cats without hematopoietic neoplasm, each T-cell subset was larger in size than in healthy cats. Finally, in cats with probable CLL the leading phenotype was CD5 + CD45R-CD4 + CD8-CD134 + MHCII+ and cell size overlapped with the one of the other diseased cats.

**Discussion:**

Our results are expected to lay the ground for a more standardized approach to feline samples for FC, and a more objective data interpretation, ultimately leading to improved diagnostic accuracy. Further studies are needed to assess the biological, diagnostic and prognostic value of specific FC patterns in feline medicine.

## 1 Introduction

Flow cytometry (FC) is widely used to diagnose and characterize lymphoma and leukemia in dogs. It allows the differentiation of reactive from neoplastic conditions by identifying a predominant homogeneous population or immunophenotypic aberrancies (1). Conversely, its use is still limited in cats because of the low availability of species-specific monoclonal antibodies and the high frequency of samples unsuitable for processing (2).

One of the most important aspects for differentiating a reactive versus a neoplastic process is the identification of cell clonality. During their development, the genes encoding the T-cell receptor gamma (TCRγ) and Immunoglobulin heavy chain receptor (IgH) undergo rearrangement. Thus, virtually each rearrangement is unique to individual lymphocytes. In a reactive population, a polyclonal expansion would be observed, while a single or predominant rearrangement indicates expansion of a population with the same antigen receptor gene. In cats, identification of clonality of TCRγ or IgH is currently performed by PCR for Antigen Receptor Rearrangement (PARR) (3–5). However, while all neoplasms are clonal in origin, not all clonal lymphocyte expansions are neoplastic and can be secondary to infectious or inflammatory diseases.

In this scenario, FC analysis of lymphoid population could be a support in differentiating truly neoplastic samples. Unfortunately, information about immunophenotypic characteristics of lymphoid subsets using FC in cats is limited, with few reports carried out in peripheral blood (PB) (6–8) and in lymph nodes (LN) (9, 10). In particular, currently there is no reference information on lymphoid subsets in PB of healthy cats, other than the percentage of CD4 + and CD8 + cells (6, 7). A deep knowledge of the composition and FC features of lymphoid circulating cells in normal and pathological conditions other than lymphoid neoplasms is mandatory, for a better interpretation of the data obtained on routine diagnostic samples, ultimately leading to a more accurate diagnosis.

The aim of this study is to describe FC features of lymphoid subsets in PB from healthy and diseased cats, in terms of percentage of different populations, cell size and degree of antigen expression, in order to provide reference information possibly useful for differentiating between reactive and neoplastic processes.

## 2 Materials and methods

### 2.1 Sample collection

All samples included in the present study had been delivered to the laboratory of clinical pathology of the Veterinary Teaching Hospital (VTH, University of Milan) after being sampled for diagnostic purposes or routine health check, with a written informed consent of the owner. Thus, specific approval of the Ethical Committee to use leftover specimens for research purposes was not required (EC decision dated 29 October 2012, renewed under protocol 02–2016, University of Milan).

Feline PB samples collected into EDTA tubes and delivered to the laboratory between January and March 2025 were considered eligible if fulfilled the following criteria: (1) adequate blood-to-EDTA ratio and lack of visible clots; (2) complete blood count (CBC) performed with an automated laser-based hematology analyzer (Sysmex XN-1000 V, Sysmex Corporation, Kobe, Japan) and blood smear evaluation; (3) availability of full signalment data and clinical records at the time of sampling; (4) negative result of a snap-test for FIV and FeLV infection at the time of sampling or within the last month. Different snap-test had been used, based on the referring veterinarian preferences. Still, all of them detected FIV antibodies and FeLV antigens.

Thereafter, samples were subdivided into healthy and diseased cats according to the criteria listed below.

Healthy cats fulfilled all the following additional criteria: (1) were sampled for routine health check or within a pre-anesthetic protocol for elective orchiectomy or ovariectomy; (2) were in good nutritional condition (Body Condition Score 4–6/9); (3) were asymptomatic and were not receiving treatments at the time of sampling; (4) more than 2 months had passed before last vaccine administration; (5) CBC and biochemistry results were within normal limits.

Diseased cats were further subdivided into two groups, based on the underlying disease.

Group 1 - diseased cats had any disease except hematopoietic neoplasia. So, they were retained in the study if: (1) had no solid lesion compatible with lymphoid neoplasia and (2a) a final diagnosis other than hematopoietic neoplasia could be reached within few days from sampling or (2b) PARR gave polyclonal results for both TCRγ and IgH. PARR was performed on WBC pellets according to already published protocols (5, 10, 11).

Group 2 - diseased cats had PB infiltration from lymphoid neoplasia, based on: (1) a single lymphoid subset prevalent at FC analysis; and (2) monoclonal result for at least one gene at PARR analysis.

### 2.2 Flow cytometry

FC was performed on EDTA PB samples within 24 h from sampling, according to already published protocols for cats (12). The antibody panel (Table 1) had been designed prior to study initiation, selecting antibodies commercially available that had already been used on feline samples in the literature or were reported to be cross-reactive with feline antigens from the manufacturer. In this latter case, cross-reactivity was preliminarily tested on random feline PB samples from the laboratory routine activity, and antibodies were included in the panel only if positive signals were obtained on the expected population, and not on the other ones. Otherwise, the antibody clone was not included in the panel, and therefore not used in the present study. All antibodies and cocktails had been titered to select the best working dilution before study initiation.

**Table 1:** Details of antibodies used to characterize circulating lymphoid subsets in 20 cats via flow cytometry.

Antigen	Expected specificity	Clone	Conjugation	Source
CD5	T-cells	f43	PE	SouthernBiotech, Birmingham, AL, USA
CD21	B-cells	CA2.1D6	AlexaFluor647	Bio-Rad, Oxford, UK
CD45R	B- and T-cells subsets	RA3-6B2	FITC	Bio-Rad
CD4	T-helper cells	3-4F4	FITC	SouthernBiotech
CD8	T-cytotoxic cells	fCD8	PE	SouthernBiotech
CD134	Activated lymphocytes	7D6	AlexaFluor647	Bio-Rad
CD9	Activated lymphocytes, platelets	MM2/57	PE	Bio-Techne, Milan, Italy
MHCII	Monocytes and lymphocytes	169-1B5.2	AlexaFluor488	Bio-Techne
CD18	All leukocytes	CA1.4E9	AlexaFluor647	Bio-Rad
CD44	All leukocytes	IM7	FITC	BD Pharmingen, San Diego, CA, USA

Five different tubes were prepared, and an adequate volume of PB was put into each of them, in order to have 500,000 cells/tube, based on instrumental WBC count. In all tubes, 25 μL of a blocking solution containing fetal bovine serum (FBS) were added, to prevent aspecific antibody binding. Thereafter, antibody cocktails were added to the five tubes, as detailed in Table 2. 7-Amino-Actinomycin D (7-AAD, BD Pharmingen, San Diego, CA, USA) was included in each sample to check cells viability. After 10 min of incubation at room temperature, 1 mL of a red blood cells lysis buffer was added to each tube. Once the sample had become transparent, a washing step was performed by centrifugation at 277 g for 8 min, the supernatant discarded, and the cell pellet resuspended in 500 μL of PBS for final acquisition. Unfortunately, Tube D could not be tested in a subset of cats due to temporary lack of the anti-MHCII antibody.

**Table 2:** Antibody cocktails used to characterize circulating lymphoid subsets in 20 cats via flow cytometry.

Tube	Fluorescence channel		
	FL1 (530/30)	FL2 (585/40)	FL4 (660/20)
Unstained	None	None	None
A	CD45R	CD5PE	CD21
B	CD4	CD8	CD134
C	CD44	CD9	CD18
D	MHCII	none	CD21

All samples were acquired with a BriCyte E6 flow cytometer (Mindray, Shenzhen, China) equipped with 2 lasers (blue and red) and 4 fluorescence channels and analyzed with the specific software MRFlow (Mindray). For each tube, 10,000 nucleated cells were acquired. For each sample from healthy cats and for samples from Group1 diseased cats, the percentage of the following lymphoid subsets was recorded: CD5 + CD45R+, CD21 + CD45R+, CD5 + CD45R-, CD4 + CD8-, CD4-CD8+, CD4 + CD8+, CD134+, MHCII+CD21+, MHCII+CD21- (Supplementary material 1). The percentages were then coupled with the absolute lymphocyte count obtained with instrumental WBC count and blood smear evaluation, to calculate the absolute concentration of each subset. In addition, for each subset, the Median Fluorescence Index (MFI) of antibody-positive cells was recorded, as well as the median forward-scatter (FSC-H) value. These parameters were not recorded for CD4 + CD8 + subset, due to the low number of cells with this phenotype. In order to allow comparison with data obtained on other instruments, for each antigen an MFIratio was calculated, by dividing the MFI of antibody-positive cells to the one of unstained lymphoid cells in the same fluorescence channel and regarded as an indicator of the degree of antigen expression (12). Pairwise, the FSC-H value of each subset was normalized with a ratio with the FSC-H value of granulocytes in the same sample (nFSC), to quantify cell size (13). Finally, CD18-MFIratio and CD44-MFIratio were calculated for the lymphoid population as a whole. Concerning Group2 samples, the aforementioned parameters were recorded setting a gate to include only neoplastic cells.

### 2.3 Statistical analyses

All data were inserted in an electronic datasheet, and descriptive statistics were calculated. A Friedman test for paired samples was performed to compare nFSC values among subsets within the group of healthy cats and within Group1 diseased cats, using Bonferroni correction for post-hoc comparisons. Wilcoxon test was used to assess possible differences in CD5-MFIratio between CD5 + CD45R + and CD5 + CD45R- cells, CD45R-MFI ratio between CD5 + CD45R + and CD21 + CD45R + cells, and MHCII-MFIratio between MHCII+CD21 + and MHCII+CD21- cells within healthy cats and within Group1 diseased cats.

Mann–Whitney test was used to assess possible differences in nFSC and MFIratio values of each lymphoid subset between healthy and Group 1 diseased cats.

Only descriptive data are presented for Group 2 diseased cats, due to low caseload and variable phenotype among cases.

All analyses were performed with SPSS Statistical software for Windows, v29.0. Significance was set at *p* ≤ 0.050.

## 3 Results

Overall, 20 feline PB samples were included in the present study, including 9 (45%) healthy cats, and 11 (55%) diseased cats (6 in Group1 and 5 in Group2).

Based on 7-AAD staining, all samples had ≥97.0% vital cells.

### 3.1 Healthy cats

Healthy cats included 8 (88.9%) Domestic Short Hair (DSH) and 1 (11.1%) Maine coon. Five (55.6%) were female (1 spayed) and 4 (44.4%) were males (1 neutered). Median age was 1 year (range: 6 months to 8 years).

Descriptive statistics of FC data are presented in Table 3. Overall, CD5 + CD45R- was the most numerous subset, followed by CD21 + CD45R+, whereas CD5 + CD45R + cells were less represented. Considering CD4 and CD8 expression, CD4 + CD8- cells outnumbered CD4-CD8+, whereas CD4 + CD8 + cells were not detected in 1 (11.1%) case and accounted for <1% in the other 8 (88.9%) cases. CD134 + cells were poorly represented, never exceeding 1,000 cells/μL. Finally, MHCII+ cells were more commonly CD21- than CD21+. No MHCII-CD21 + cell was detected in any sample. Tube D (Table 2) was not tested in 1 case.

**Table 3:** Flow cytometric features of lymphoid subsets in 9 healthy cats.

Cell subset	Parameter	Median	Range
All lymphocytes	Count (cells/μL)	4,564	2,697–5,384
	nFSC	0.60	0.57–0.98
	CD18-MFIratio	37.65	24.17–157.11
	CD44-MFIratio	134.80	107.34–179.32
CD5 + CD45R-	Count (cells/μL)	1,586	648–3,136
	nFSC	0.62	0.57–0.69
	CD5-MFIratio	545.63	391.76–709.68
CD21 + CD45R+	Count (cells/μL)	1,080	421–2,405
	nFSC	0.63	0.59–0.66
	CD21-MFIratio	202.23	98.37–698.11
	CD45R-MFIratio	66.38	32.16–145.41
CD5 + CD45R+	Count (cells/μL)	134	65–439
	nFSC	0.64	0.61–0.69
	CD5-MFIratio	312.86	83.42–388.46
	CD45R-MFIratio	28.56	13.51–66.83
CD4 + CD8-	Count (cells/μL)	1,260	393–1783
	nFSC	0.60	0.56–0.67
	CD4-MFIratio	61.67	34.31–82.44
CD4-CD8+	Count (cells/μL)	429	143–548
	nFSC	0.61	0.56–0.66
	CD8-MFIratio	219.24	141.51–430.81
CD4 + CD8+	Count (cells/μL)	7	0–29
CD134+	Count (cells/μL)	198	54–569
	nFSC	0.67	0.59–0.74
	CD134-MFIratio	61.20	21.92–134.59
MHCII+CD21+	Count (cells/μL)	1,078	314–2061
	nFSC	0.63	0.59–0.67
	MHCII-MFIratio	294.20	164.30–514.78
MHCII+CD21-	Count (cells/μL)	1,574	960–2,788
	nFSC	0.63	0.58–0.68
	MHCII-MFIratio	27.55	18.95–76.01

nFSC = ratio between median forward scatter (FSC-H) value of the population of interest and granulocytes in the same sample; MFIratio = ratio between median fluorescence index of stained and unstained cells.

All lymphoid cells were smaller in size than granulocytes, with a median nFSC ranging from 0.60 to 0.67. Significant differences among subsets were found (*p* = 0.002), with CD134 + cells being larger than both CD4 + CD8- and CD4-CD8 + cells (*p* = 0.037 for both post-hoc analyses).

**Figure 1:**
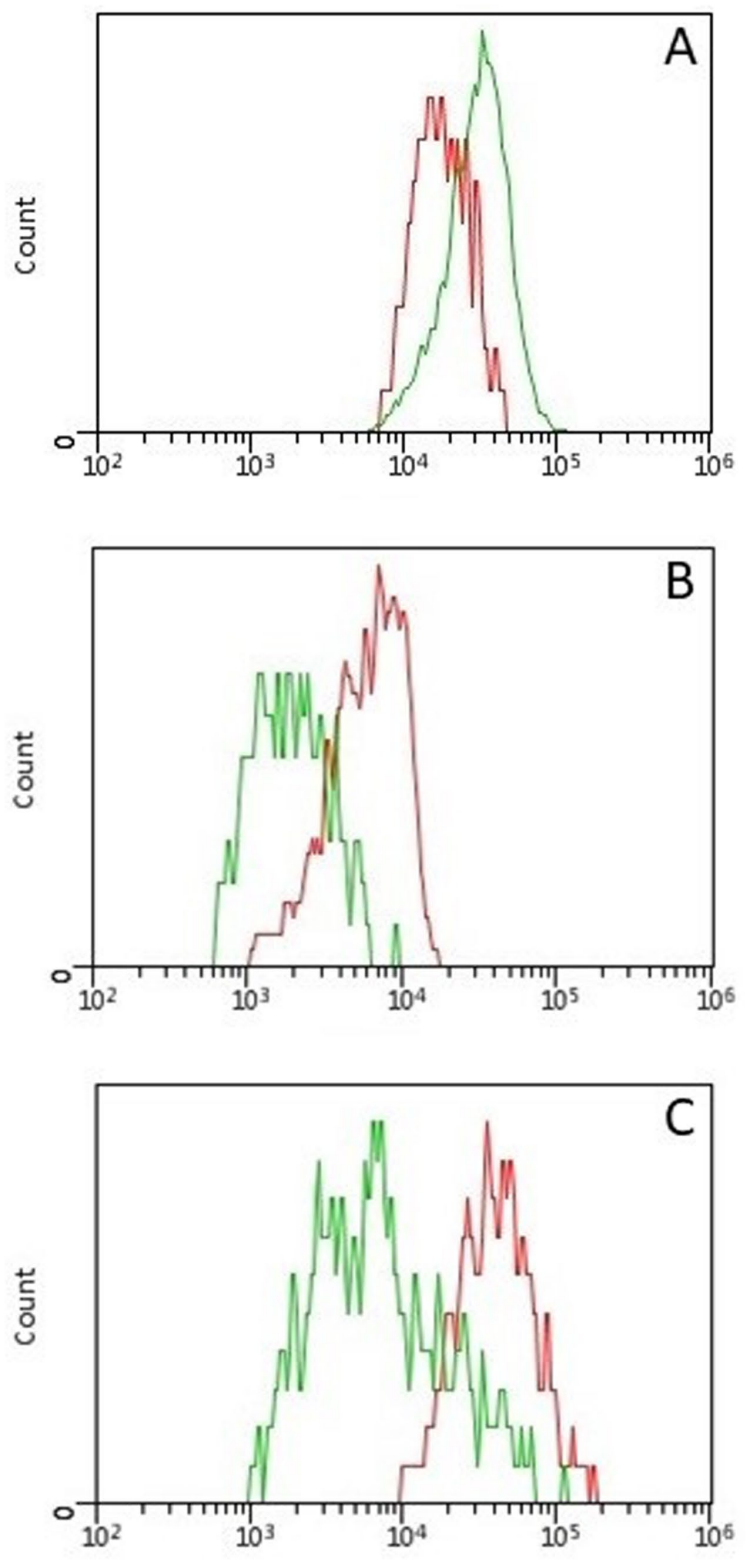
Histogram overlays showing differences in fluorescence intensity between lymphoid subsets in 9 healthy cats. All differences were statistically significant (*p* < 0.05) **(A)** Fluorescence intensity of CD5 was higher in CD5 + CD45R- (green line) than in CD5 + CD45R + (red line) cells. **(B)** Fluorescence intensity of CD45R was higher in CD21 + CD45R + (red line) than in CD5 + CD45R + (green line) cells. **(C)** Fluorescence intensity of MHCII was higher in MHCII+CD21 + (red line) than in MHCII+CD21- (green line) cells.

Concerning MFI analyses, CD5-MFIratio was higher in CD5 + CD45R- than in CD5 + CD45R + cells (*p* = 0.008), CD45R-MFIratio was significantly higher in CD21 + CD45R + than in CD5 + CD45R + cells (*p* = 0.008), and MHCII-MFIratio was higher in MHCII+CD21 + than in MHCII+CD21- cells (*p* = 0.012). These data are represented in Figure 1.

No CD44 + CD9 + CD18 + cell was detected in any sample. However, variable amounts of CD9 + events were present among samples. When back-gating that population of cells, it appeared to be mostly distributed in the low-left angle and along the diagonal of the morphological scattergram (Supplementary material 2). Those events were thus considered possible platelet clumps and were not further analyzed.

### 3.2 Diseased cats without hematopoietic neoplasia (Group 1)

The 6 cats in Group 1 included 5 (83.3%) DSH and 1 (16.7%) Chartreux. There were 5 (83.3%) male (2 neutered) and 1 (16.7%) female. Median age was 1.5 years (range: 8 months to 13 years). They were diagnosed with: hypertrophic cardiomyopathy, chronic kidney disease, lung carcinoma, dermatitis, trauma from car accident, and dental abscess (1 each). No PARR analysis was needed, since none of them had a clinical suspicion of lymphoid neoplasia, nor a prevalent lymphoid subset at FC.

Descriptive statistics of FC data are presented in Supplementary material 3.

Overall, the relative prevalence of cell subsets overlapped the one encountered in healthy cats. CD4 + CD8 + cells were not detected in 1 (16.7%) case, accounted for <1% in 4 (66.7%) cases, and for 1.28% in 1 (16.7%) case. CD134 + cells never exceeded 1,000 cells/μL. Tube D (Table 2) was tested only in 3 cats. Among them, MHCII+ cells were more commonly CD21- than CD21+. No MHCII-CD21 + or CD44 + CD9 + CD18 + cell was detected in any sample.

All lymphoid cells were smaller in size than granulocytes. Significant differences in nFSC were found among subsets (*p* = 0.008). At post-hoc analyses, only CD134 + cells were significantly larger in size than CD21 + CD45R + cells (*p* = 0.002).

Concerning MFI analyses, CD5-MFIratio was significantly higher in CD5 + CD45R- than in CD5 + CD45R + cells, and CD45R-MFIratio was significantly higher in CD21 + CD45R + cells than in CD5 + CD45R + cells (*p* = 0.028 for both analyses).

**Figure 2:**
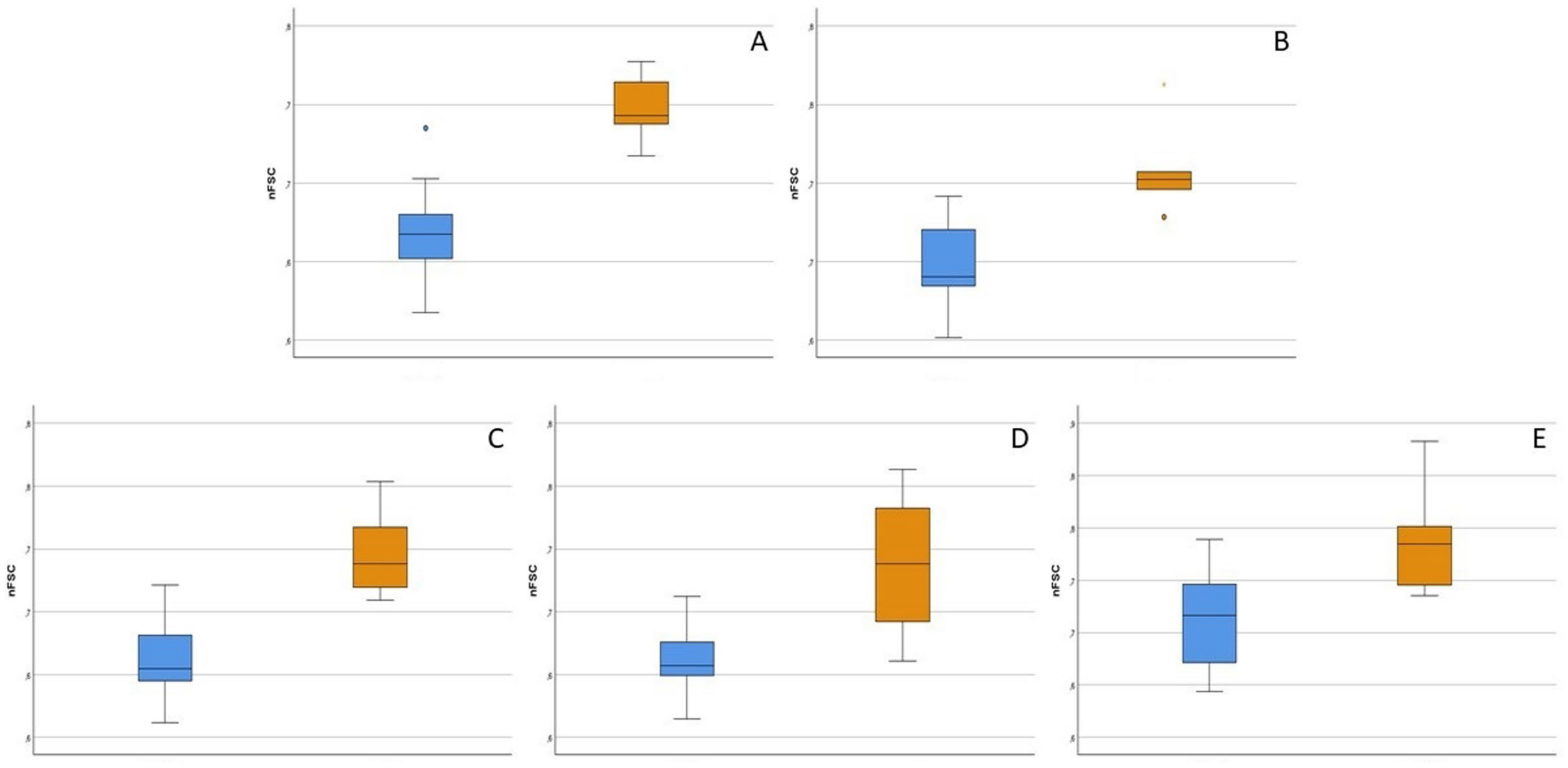
Box-plot showing flow cytometric features of lymphoid subsets that significantly differed between 9 healthy (light blue boxes) and 6 diseased without hematopoietic neoplasia (orange boxes) cats (*p* < 0.05). nFSC = ratio between median forward scatter (FSC-H) value of the population of interest and granulocytes in the same sample. For each box, the main horizontal line represents the median value, the lower and upper limits of the box represent the first and third quartile, the box represents the interquartile range, and the upper and lower whiskers represent the upper and lower value that is not an outlier. **(A)** nFSC of CD5 + CD45R- cells. **(B)** nFSC of CD5 + CD45R + cells. **(C)** nFSC of CD4 + CD8- cells. **(D)** nFSC of CD4-CD8 + cells. **(E)** nFSC of CD134 + cells.

The parameters for which a significant difference between healthy cats and Group1 diseased cats was found, are shown in Figure 2. In particular, CD5 + CD45R + and CD5 + CD45R- cells were larger in size in reactive samples (*p* = 0.002 for both analyses), as well as CD4 + CD8- (*p* = 0.003), CD4-CD8 + (*p* = 0.013) and CD134 + cells (*p* = 0.018). Conversely, no difference in cell counts or MFIratios was found.

### 3.3 Diseased cats with lymphoid neoplasia (Group 2)

**Figure 3:**
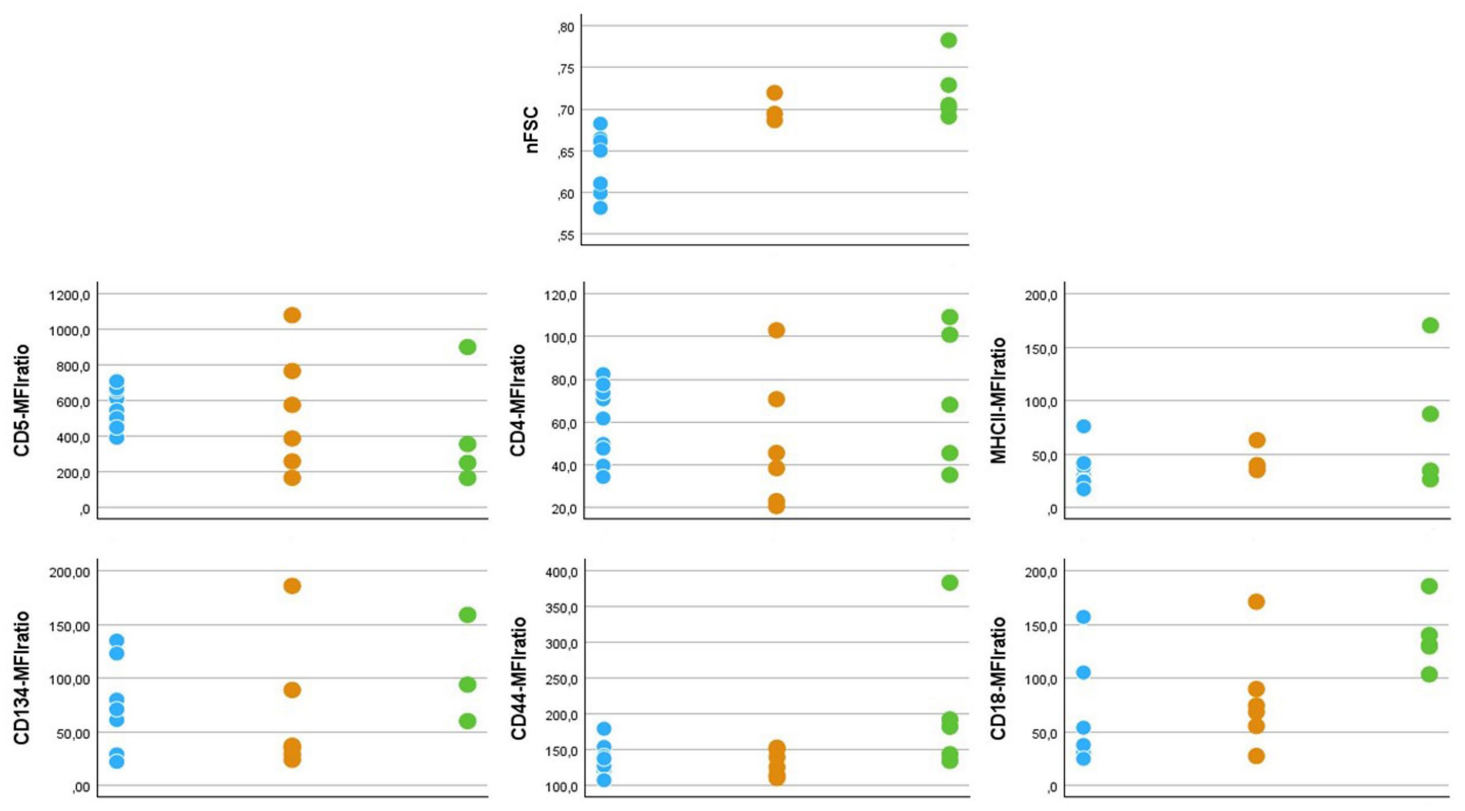
Dot graphs showing flow cytometric features of T-cell subsets in 9 healthy (light blue dots), 6 diseased without hemaopoietic neoplasia (orange dots) and 5 diseased with chronic lymphocytic leukemia (green dots) cats. nFSC = ratio between median forward scatter (FSC-H) value of the population of interest and granulocytes in the same sample. Since all neoplastic samples had a MHCII+CD21- phenotype, nFSC values were taken from this population also in healthy and diseased cats without hematopoietic neoplasia, in order to allow comparison. MFIratio = ratio between antibody-stained and unstained cells in the same fluorescence channel.

All 5 cats diagnosed with lymphoid neoplasia were DSH. Three (60%) were female (2 spayed) and 2 (40%) were male (1 neutered). Median age was 11 years (range: 9 to 17 years). All of them presented with mature lymphocytosis as the sole abnormality. Median lymphocytes count was 67,525 cells/μL (range: 17314 to 691,564 cells/μL). Diagnostic workup and staging procedures varied among cases, but solid lesions were never detected. A prevalent T-cell population was detected in FC and clonal TCRγ rearrangement was confirmed by PARR, thus leading to a probable diagnosis of T-cell chronic lymphocytic leukemia (T-CLL). The phenotype of neoplastic cells was CD5 + CD45R-CD4 + CD8-CD134 + MHCII+ in 3 cases, CD5 + CD45R-CD4 + CD8-CD134-MHCII+ in 1 case, and CD5-CD45R-CD4 + CD8-CD134-MHCII+ in 1 case. In all cases, neoplastic cells stained positive for CD44 and CD18, and negative for CD21 and CD9. Raw data of nFSC and MFIratios of antibody-positive neoplastic cells are represented in Figure 3, together with those of the respective cellular subsets in healthy cats and Group1 diseased cats.

## 4 Discussion

To the authors knowledge, this is the first study describing FC features of circulating lymphoid subsets in healthy cats using a 10 antibody panel. Data on diseased cats with and without lymphoid neoplasia are also presented.

Flow cytometry is only at its debut in feline oncology. In the past, indeed, it was mentioned in the diagnostic workup of occasional case reports (14, 15), while studies specifically focused on FC analysis of oncological samples date back to less than one decade ago (2, 8, 9, 16, 17). Importantly, data on non-neoplastic cells in cats are limited to two recent studies (10, 12). Herein, we applied a large antibody panel and report a number of FC features for each lymphoid cell subset in healthy cats, including absolute count, cell size (expressed as nFSC) and degree of antigen expression (expressed as MFIratio).

While designing the antibody panel, we encountered some unexpected findings with CD9 antibody, which prevented us from confirming the cross-reactivity with feline antigen reported by the manufacturer. Still, we retained the antibody in the panel since we believe that our findings could help improve FC analysis of feline samples, irrespective of the precise antigen detected by the antibody. According to the published literature, CD9 should be expressed on all leukocytes and platelets (18–20). However, we detected no CD9 + leukocyte. Unfortunately, CD9 expression has never been tested on feline fresh PB samples. Thus, we could not define whether our findings are linked to the antibody clone or to minimal to null CD9 expression on circulating feline WBC. However, we found a variable amount of CD9 + events among samples and among tubes within single samples. Better separation of WBC subclasses was obtained in the morphological scattergram when excluding those CD9 + events (Supplementary Figure 2). This fact, together with the peculiar distribution of CD9 + events in the morphological scattergram, and concomitant negative staining for the panleukocyte markers CD44 and CD18, prompted us to speculate that those events could represent platelet clumps. Further studies are needed to confirm this hypothesis.

Overall, three main populations were identifiable in healthy subjects and Group1 diseased cats, namely CD5 + CD45R-, CD21 + CD45R + and CD5 + CD45R+, in decreasing order of prevalence. CD45R has been used in the literature to identify B-cells in cats (21, 22). However, its expression on effector T-cytotoxic cells and neoplastic T-cell has also been reported (23–25). Our results suggest that CD45R expression is not limited to B-cells even in normal conditions. Concomitant use of additional B- and T-cell markers is necessary to accurately identify lymphoid subsets. Additionally, analysis of CD45R- and MHCII-MFIratios could be of aid in discriminating among the 3 subclasses, based on the significant differences we reported, in both healthy and Group1 diseased cats. Unfortunately, we did not couple CD45R with further T-cell markers, such as CD4 and CD8. Thus, the full antigenic pattern of CD5 + CD45R + cells and their the biological role still have to be defined.

When comparing healthy and Group1 diseased cats, an increase in the cell size of all T-cell subsets was noted. This could be attributed to their activation, although not all cats had inflammatory/infectious diseases. Irrespective of the underlying mechanisms, this difference is relevant from a diagnostic point of view, since it remarks that moderate increase in T-cell size alone should not be considered a criterium for diagnosing lymphoid neoplasia in cats.

When considering CD4 and CD8 expression, herein we confirmed the higher prevalence of CD4 + CD8- cells already reported in healthy cats in literature (6, 26). More interestingly, we reported a small amount of CD4 + CD8 + cells in >80% samples, without differences between healthy and Group1 diseased cats. It seems reasonable to argue that in the two remaining cats (1 healthy and 1 diseased cat) CD4 + CD8 + cells count was lower than the detection limit of the technique, rather than being truly absent. In the literature, low percentages of CD4 + CD8 + cells have been found in normal or reactive lymph nodes in cats (10), whereas higher proportions can be found in mediastinal masses. This is due to the fact that CD4 + CD8 + cells represent a transient stage during T-cell maturation (27). The percentage of CD4 + CD8 + cells in mediastinal masses is a leading criterium for FC differential between mediastinal lymphoma and thymoma in dogs, but the same is not valid in cats (16, 28). Altogether, our results and the data already published suggest that CD4 + CD8 + phenotype should not be considered a hallmark of neoplasia in cats.

We also found a low number of CD134 + cells in circulation without differences between healthy and Group1 diseased cats. CD134, also named OX40, is a member of the tumor necrosis factor (TNF) receptor superfamily, and works as a co-stimulator of lymphocyte activation, preventing apoptosis and enhancing proliferation (29, 30). In cats, this molecule is considered a key-driver of the progression of FIV infection, although almost all studies on this topic report in-vitro results (31–33). All cats included in the present study tested negative for FIV antibodies within the last month prior to sampling. Thus, in the future, it could be interesting to test the concentration of CD134 + cells in FIV-infected cats with different stage of disease, in order to assess its possible clinical relevance in the management of such patients. In addition, 3 out of 5 neoplastic samples stained positive for CD134. To the authors’ knowledge, this is the first report of CD134 expression in neoplastic cells in cats. Thus, it is still unknown whether it reflects a different stage of maturation of the cells, or has any prognostic implication.

Concerning the data from 5 cats diagnosed with probable T-CLL we present herein, T-cell lineage was confirmed in all cases by PARR TCRγ monoclonal results and FC expression of CD4. Our results agree with a recent study, which reported a high prevalence of CD4 + neoplastic lymphocytosis in cats, whereas B-cell lymphocytosis was generally not clonal, and indeed not represented in our caseload (8). Of notice, Rout and colleagues also identified a less common “CD5low” subgroup, bearing a worse prognosis. In contrast, the majority of the cases in the present study (3 out of 4 CD5+) had lower CD5-MFIratio than normal T-cells, and one case was even CD5-. Unfortunately, we lack follow-up data for our cohort, thus preventing comparison with the survival data for the “CD5low” disease.

Neoplastic cells were similar to those of the non-neoplastic counterparts, either in healthy or Group1 diseased cats, with the only exception of CD5-MFIratio (Figure 3). In particular, nFSC values overlapped in Group1 and Group2 diseased cats (Figure 3). Once more, this fact suggests that cell size alone should not be used to differentiate between neoplastic and non-neoplastic cells in cats. Another interesting finding is the level of expression of CD18. We already reported that this integrin is expressed at low levels in circulating lymphocytes in healthy cats (12). Based on our present results, CD18 seems to be overexpressed on neoplastic cells. On one side, this could play a role in the spread of the disease, since this molecule is involved in leukocyte rolling and extravasation (34). On the other hand, the differential degree of expression could serve as a laboratory tool to be included in the diagnostic process for suspected feline leukemias. Further studies are needed to support both hypotheses.

The low number of samples included represent the main limitation of the present study. This is particularly true for diseased cats, where subjects with different diseases were included in Group1, and all Group2 cats had a T-cell neoplasia. Also, the cats included had different breed, sex and age possibly biasing our results. Because of this, they should not be interpreted as reference interval for healthy cats or laboratory parameters to differentiate among clinical conditions. Rather, they represent a basis to improve FC analysis of feline samples in a diagnostic setting. Finally, the lack of follow-up data for cats with probable CLL prevented us from making any hypothesis about possible prognostic role of the different phenotypes we recorded.

In conclusion, this is the first study reporting FC features of lymphocyte subsets in cats, applying a large antibody panel, and highlighting some differences between healthy and diseased cats, either with neoplastic or non-neoplastic conditions. The antibody panel we present is composed of fluorochrome-conjugated antibodies available in the market, and as such can be easily reproduced in any veterinary FC facility. However, it is important to note that some of the antibody clones we used are reported not to work on preserved samples (35). Thus, the panel is suitable only to analyze fresh samples, which is overall generally recommended in FC practice. Future studies should address the biological and diagnostic relevance of FC data in cats.

## Data Availability

The raw data supporting the conclusions of this article will be made available by the authors, without undue reservation.
