# Supplementary material for: Flow cytometric features of lymphoid subsets in healthy and diseased cats
Source: Front Vet Sci. 2025 Aug 1;12:1640229. doi: 10.3389/fvets.2025.1640229 (PMC12353732; doi:10.3389/fvets.2025.1640229)

Supplementary Figure 1: flow cytometric scattergrams showing the gating strategy used to characterize circulating lymphocytes subsets in 15 cats. **A:** all events are shown. Doublets were excluded on an FSC-A versus FSC-H scattergram. **B:** only singlets are shown. A gate was set to include only nucleated cells in an FSC-H versus SSC-H scattergram. **C:** only nucleated cells are shown. A gate was set to include only lymphocytes, identified based on low FSC-H and SSC-H values. **D:** lymphocytes are shown based on CD45R and CD5 expression. Three main population are detected. Backgating revealed that the CD45R+CD5- population (right low quadrant) stained positive for CD21 (data not shown). **E:** lymphocytes are shown based on CD4 and CD8 expression. **F:** lymphocytes are shown based on MHCII and CD21 expression.

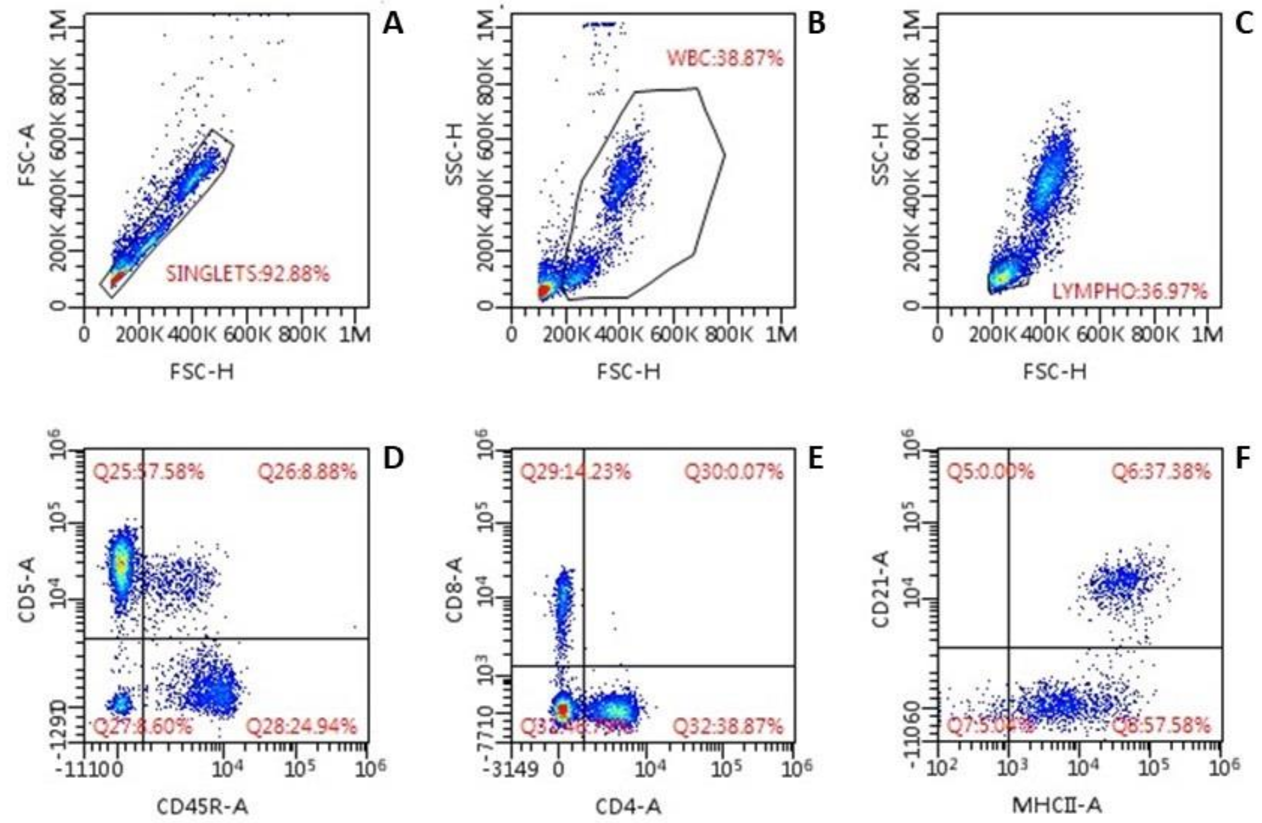

Supplement: Supplementary file 1 [file Data_Sheet_1.pdf]
