# Supplementary material for: Flow cytometric features of lymphoid subsets in healthy and diseased cats
Source: Front Vet Sci. 2025 Aug 1;12:1640229. doi: 10.3389/fvets.2025.1640229 (PMC12353732; doi:10.3389/fvets.2025.1640229)

Supplementary Figure 2: flow cytometric scattergram showing results of staining with CD9-antibody in a representative blood sample from a healthy cat. **A:** only singlets are shown in an FSC-H versus SSC-H scattergram. **B:** singlets are shown based on CD9 expression. A gate (P2) was set to include only CD9-negative events, and a second gate (P3) was set to include only CD9-positive events. **C:** only P2 events are shown in an FSC-H versus SSC-H scattergram. Leukocyte subpopulation are more easily identifiable than in panel A. **D:** only P3 events are shown in an FSC-H versus SSC-H scattergram. They are located in the low-left angle and the diagonal of the scattergram.

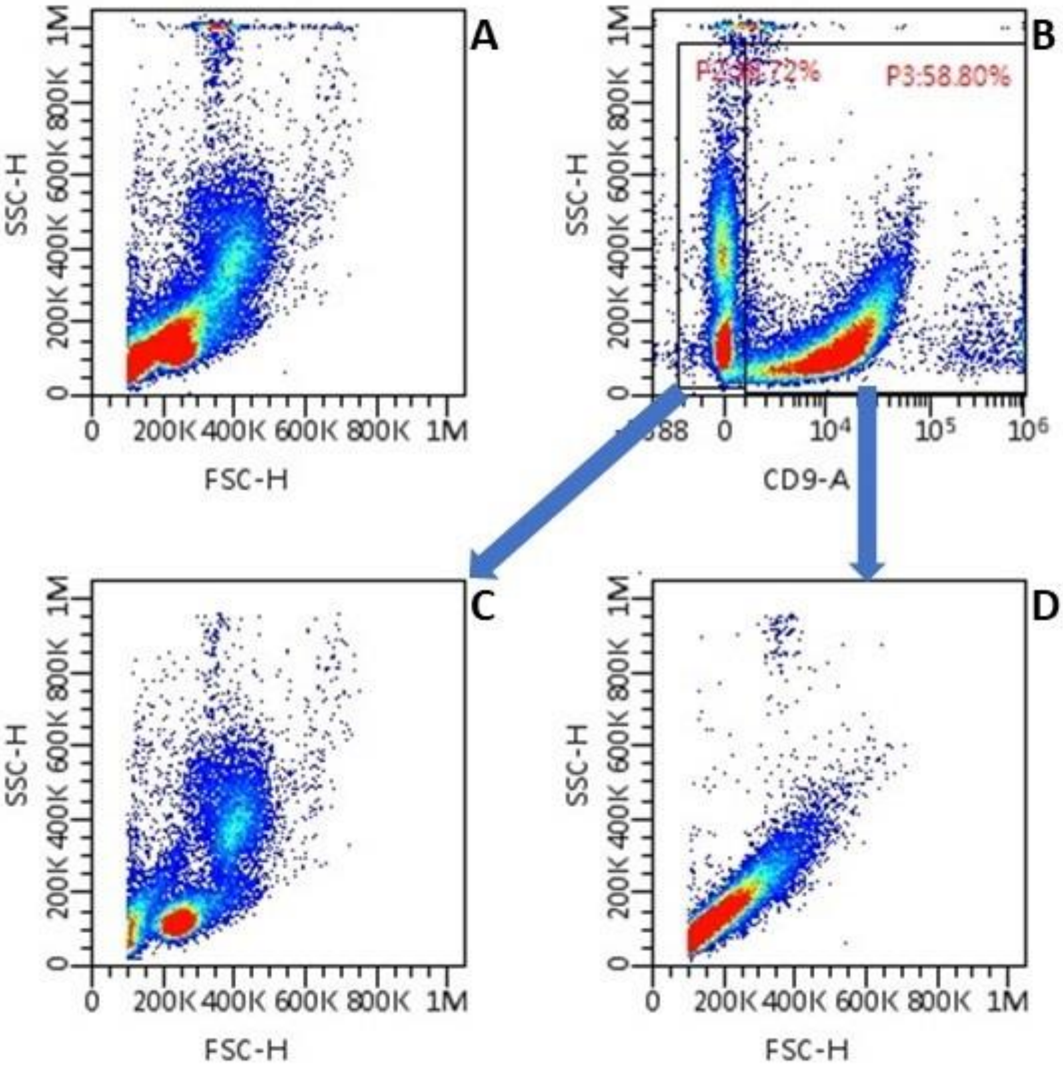

Supplement: Supplementary file 2 [file Data_Sheet_2.pdf]
