# Supplementary material for: Flow cytometric features of lymphoid subsets in healthy and diseased cats
Source: Front Vet Sci. 2025 Aug 1;12:1640229. doi: 10.3389/fvets.2025.1640229 (PMC12353732; doi:10.3389/fvets.2025.1640229)
Supplement: Supplementary file 3 [file Data_Sheet_3.pdf]

Supplementary Table 1: flow cytometric properties of lymphoid subsets in 6 diseased cats without hematopoietic neoplasia

Cell subset	Parameter	Median	Range
All lymphocytes	Count (cells/µL)	3837	2341-11654
	nFSC	0.70	0.67-0.72
	CD18-MFIratio	71.49	27.44-171.26
	CD44-MFIratio	132.59	110.40-152.78
CD5+CD45R-	Count (cells/µL)	2059	900-7897
	nFSC	0.69	0.67-0.73
	CD5-MFIratio	482.03	166.88-1080.06
CD21+CD45R+	Count (cells/µL)	715	468-909
	nFSC	0.67	0.59-0.75
	CD21-MFIratio	225.71	84.46-758.97
	CD45R-MFIratio	46.31	22.81-125.96
CD5+CD45R+	Count (cells/µL)	165	50-984
	nFSC	0.70	0.68-0.76
	CD5-MFIratio	89.05	15.31-510.39
	CD45R-MFIratio	29.08	11.29-59.90
CD4+CD8-	Count (cells/µL)	1165	246-3441
	nFSC	0.69	0.66-0.75
	CD4-MFIratio	41.96	20.64-102.94
CD4-CD8+	Count (cells/µL)	411	206-3063
	nFSC	0.69	0.61-0.76
	CD8-MFIratio	218.03	66.93-333.33
CD4+CD8+	Count (cells/µL)	13	0-149
CD134+	Count (cells/µL)	254	53-528
	nFSC	0.73	0.69-0.83
	CD134-MFIratio	36.05	23.61-186.17
MHCII+CD21+*	Count (cells/µL)		954; 972; 1208
	nFSC		0.66; 0.69; 0.72
	MHCII-MFIratio		286.57; 376.41; 393.33
MHCII+CD21-*	Count (cells/µL)		1672; 2198; 9371
	nFSC		0.69; 0.69; 0.72
	MHCII-MFIratio		34.98; 39.73; 62.99

nFSC= ratio between median forward scatter (FSC-H) value of the population of interest an granulocytes in the same sample; MFIratio = ratio between median fluorescence index of stained and unstained cells.
*=data obtained in 3 samples only
